# Classification of sporadic Creutzfeldt-Jakob disease based on resting state scalp-recorded electroencephalogram-derived indices

**DOI:** 10.1371/journal.pone.0355367

**Published:** 2026-08-06

**Authors:** Chisho Takeoka, Tetsushi Yada, Toshimasa Yamazaki, Yoshiyuki Kuroiwa, Toshiaki Hirai, Kimihiro Fujino, Hidehiro Mizusawa, Masaki Takao, Yasuo Terao, Masahito Yamada

**Affiliations:** 1 Graduate School of Computer Science and Systems Engineering, Kyushu Institute of Technology, Fukuoka, Japan; 2 University Hospital, Mizonokuchi Teikyo University School of Medicine, Kanagawa, Japan; 3 National Center of Neurology and Psychiatry, Tokyo, Japan; 4 Faculty of Medicine, Kyorin University, Tokyo, Japan; 5 Department of Neurology, Kudanzaka Hospital, Tokyo, Japan; The University of Texas Health Science Center at Houston, UNITED STATES OF AMERICA

## Abstract

Prion disease is a general term for a disease that causes cognitive disorders due to the accumulation of abnormal prion protein in the brain. Creutzfeldt-Jakob disease (CJD) is the most common case of prion disease, and sporadic Creutzfeldt-Jakob disease (sCJD) accounts for more than 70% of CJD cases. Early and accurate diagnosis of sCJD remains challenging. The aim of this study is to classify 6 sCJD patients from 10 healthy older adults and 23 Alzheimer’s disease (AD) patients using resting-state scalp-recorded electroencephalogram (EEG)-derived indices. Power spectrum, SL values by Synchronization Likelihood (SL), and graph metrics by SL values were calculated for 5 frequency bands as EEG-derived indices. In addition, power spectrum and SL values were standardized and exponentially transformed for each subject and each frequency band. Graph metrics were calculated by these SL values. These indices were used as features for classification. Classifiers were constructed by features selected by Recursive Feature Elimination (RFE). The highest classification accuracy was 97.44% using a 12-dimensional feature. This accuracy was confirmed by indices after standardization and exponential transformation. Additional validation analyses were performed to assess the reliability of the selected classifier. Accuracy of nested LOOCV was 84.62%, supporting meaningful classification ability under a leakage-controlled validation framework. An analysis of robustness removing a group of subjects with high similarity with many others showed that the selected classifier maintained a micro-F1 score of 90.32%. Permutation test indicated that the observed performance was significantly higher than chance level, and repeated stratified 10-fold cross-validation showed relatively stable performance across different data partitions. These findings suggest that resting-state EEG-derived indices may provide useful candidate features for classification of sCJD, AD, and healthy older adults. However, further validation using larger independent cohorts is required to establish the generalizability and clinical reliability of the proposed classifier.

## 1. Introduction

Prion disease is one of the fatal neurodegenerative diseases. It is classified under transmissible spongiform encephalopathies (TSE). The prion is formed by structural changes in the normal host protein (PrPC) and has transmissibility. The accumulation of this in the central nervous system leads to prion disease. Creutzfeldt-Jakob disease (CJD) is a representative case of human prion disease. This includes sporadic, genetic, and acquired forms, with reported cases in Japan from April 1999 to February 2021 accounting for 76.2%, 21.0%, and 2.3% respectively [1]. The incidence of prion disease is estimated to be a few cases per million people each year, but there have been reports of an increasing trend in the past [2,3].

The cases and diagnostic criteria for prion diseases are outlined in guidelines described by the Centers for Disease Control and Prevention (CDC) in the United States and the National Center of Neurology and Psychiatry in Japan [1,4]. Typical cases of CJD include rapidly progressive dementia, ataxia, visual disturbances, myoclonus, pyramidal/extrapyramidal signs, and akinetic mutism. In diagnosis of CJD, blood and urine tests, electroencephalogram (EEG), MRI scans, and cerebrospinal fluid examinations are conducted to distinguish prion diseases from other disorders such as Alzheimer’s disease.

There is no established treatment for prion diseases until now. Early and accurate diagnosis is considered a significant challenge in understanding the progression and symptoms of the disease. Additionally, Connor et al. said that obtaining a confident antemortem diagnosis of prion disease is important for infection control purposes, for excluding other difficult-to-diagnose but potentially treatable neurological diseases, and for helping to prepare the patient and loved ones for end-of-life care [5].

Several studies have been reported with the goal of contributing to highly accurate antemortem diagnosis. Bizzi et al. validated the diagnostic performance of a new diffusion MRI toward more accurate diagnosis of sporadic Creutzfeldt-Jakob disease (sCJD). Their results revealed that this method was superior to conventional MRI diagnosis and showed potential clinical significance [6]. Additionally, Orrú et al. applied real-time quaking-induced conversion (RT-QuIC) with improved analytical sensitivity to cerebrospinal fluid (CSF). Their study demonstrated high sensitivity and specificity. These results indicated the possibility of rapid and accurate ante-mortem diagnosis of CJD [7].

In this study, we will conduct analyses for establishing premortem diagnosis by EEG data. EEG is advantageous as a biomarker due to its low cost and minimal burden on subjects. Moreover, as means to evaluate the reliability of biomarkers, research has been conducted targeting high accuracy classification of various neurological disorders (Alzheimer’s disease [8], epilepsy [9,10], stroke [11], schizophrenia [12,13], Parkinson’s disease [14,15], depression [16,17], and bipolar disorder [18,19]). In our previous study, we compared with EEG-derived indices of prion disease patients, dementia patients, and healthy controls through one-way analysis of variance [20]. Morabito et al. calculated the mean, standard deviation, and skewness of wavelet coefficients obtained from continuous wavelet transform of EEG were used as features and constructed 3 classifiers for binary classification: CJD versus rapidly progressive dementia (RPD) patients, CJD versus healthy controls, and CJD versus AD patients [21].

Studies comparison or classification CJD patients using indices that can be calculated from EEG, as in these previous studies, remain at early stages. The aim of our study is construction of the classifier that enables the high accuracy classification of 3 subject groups (Healthy older adults, AD patients, and sCJD patients). To achieve this, power spectrum, SL values by Synchronization Likelihood (SL) [22,23], and graph metrics were calculated. Graph metrics were calculated by SL values. These indices were applied machine learning as features. To achieve high accuracy classification, power spectrum and SL values were standardized and exponentially transformed. Graph metrics were calculated by SL values after standardization and exponential transformation. Features were selected to construct classifiers. Feature selection is performed by Recursive Feature Elimination (RFE). RFE is a feature selection method by a machine learning algorithm. Features selected by RFE could vary depending on the algorithm. Some algorithms were applied to RFE. Additionally, the number of features to be selected by RFE can be specified. By increasing the number of features selected gradually, various combinations of features were constructed. In this study, we show that standardization and exponential transformation of indices and construction of many classifiers were the key factor constructing high accuracy classifier. Additionally, SL values and graph metrics were important indices in the classification.

## 2. Materials and methods

### 2.1. EEG measurement

Subjects consisted of 10 healthy older adults, 23 AD patients, and 6 sCJD patients. This retrospective study used EEG data obtained from patients previously diagnosed with Alzheimer’s disease or sporadic Creutzfeldt-Jakob disease at Mizonokuchi Hospital. This study was approved by the Institutional Review Board of the National Center of Neurology and Psychiatry (approval number B2024-077). The data were accessed for research purposes on 28 January 2025. The authors did not have access to information that could identify individual participants. Written informed consents were obtained from all participants. The study protocol, including all EEG data analysis, was approved by Research Ethics Committee, Faculty of Medicine, Teikyo University. All procedures performed in studies involving human participants were in accordance with the ethical standards of the 1964 Helsinki Declaration and its later amendments or comparable ethical standards. EEG measurements were performed at Mizonokuchi Hospital, Teikyo University School of Medicine, through a Nihon Kohden EEG-1224. The device was equipped with 16 Ag/AgCl electrodes (channels) (a Nihon Kohden H503A). Electrodes were attached at Fp1, Fp2, F3, F4, C3, C4, P3, P4, O1, O2, F7, F8, T3, T4, T5, and T6 based on the international 10–20 system. In addition to the 16 electrodes, A1 and A2 (reference electrodes) were attached to both the earlobes. The sampling frequency was set to 500 Hz and a low-pass filter (=120 Hz), except for the Japanese electrical power noise (50 Hz), was set to remove artifacts. All subjects were asked to lie on their backs in a resting position with eyes closed for at least 5 minutes. For all subjects, EEG data from the second to third minute of measurement was used as stable EEG data for analysis.

All EEG data were reconstructed by Fourier transform and inverse Fourier transform into 5 frequency bands: theta (θ) (4–8 Hz), lower alpha (α1) (8–10 Hz), upper alpha (α2) (10–13 Hz), beta (β) (13–30 Hz), and gamma (γ) (30–45 Hz).

### 2.2. EEG-derived indices

As EEG-derived indices, power spectrum, SL values by Synchronization Likelihood (SL) [22,23], and graph metrics were calculated.

#### 2.2.1. Power spectrum.

By calculating the band power of each frequency band for each electrode, dataset of the power spectrum was created. The values were calculated by the pspectrum function in MATLAB. In this study, 16 power spectrum values ${PS}_{i}\;(i=\text{1},\text{2},\cdots ,\text{1}\text{6})$ were calculated.

#### 2.2.2. Synchronization Likelihood (SL).

Synchronization Likelihood (SL) [22,23] was applied between all two electrodes in each of the 5 frequency bands. The SL value is a measure of synchronization between the two electrodes, which avoids bias due to the degrees of freedom of the interacting subsystems and can deal with non-stationary dynamics. This value ranges from 0 to 1, and higher SL values indicate higher synchronization. The basic principle of SL is to divide each time series into a series of “patterns” (short portions of the time series containing several cycles of the main frequency) and to search for repetitions of these patterns. For mathematical details on the calculation of SL values, see [23]. In this study, $n_{rec}=\text{1}\text{0}$ and $p_{ref}=\text{0}.\text{0}\text{1}$. To calculate SL time series, reference time i increases with 32 ms increment. The first and last data points of SL time series were deleted (40 data points). SL value is the average of SL time series after deletion of data points. SL values of ${{\;}_{\text{1}\text{6}}C}_{\text{2}}$=120 pairs of electrodes were calculated because EEGs were recorded at 16 electrodes. In this study, 120 SL values $SL_{ij}\;(i<j;\;i,\;j=\text{1},\text{2},\cdots ,\text{1}\text{6}))$ were calculated.

#### 2.2.3. Standardization and exponential transformation.

16 power spectrum values ${PS}_{i}\;(i=\text{1},\text{2},\cdots ,\text{1}\text{6})$ were standardized and exponentially transformed as $exp(Z({PS}_{i})$). In addition, 120 SL values $SL_{ij}\;(i<j;\;i,\;j=\text{1},\text{2},\cdots ,\text{1}\text{6})$ were standardized and exponentially transformed as $exp(Z(SL_{ij})$). Standardization was applied to normalize the relative distribution of power spectrum and SL values within each subject and each frequency band. Exponential transformation was then used to convert all standardized SL values into positive values. This transformation enabled construction of non-negative weighted matrices suitable for calculation of graph metrics. In addition, it also maintained consistency in the feature scaling of SL values and power spectrum.

#### 2.2.4. Graph metrics.

Based on undirected weighted matrix A (elements of A; $a_{ij}$) consisted of $SL_{ij}$ or$exp(Z(SL_{ij}))$, graph metrics (vertex strength, clustering coefficient, characteristic path length, efficacy, small-worldness, modularity, eigenvector centrality, hub centrality, closeness centrality, and PageRank) were calculated. Graph metrics were calculated for each frequency band. Path length between electrodes i and j was defined as $\frac{\text{1}}{a_{ij}}$.

Vertex strength (V) [24] measures the strength of vertices in terms of the total weight of their connections.


$$\begin{array}{c} V=\;\frac{\text{1}}{N}\sum_{i=\text{1}}^{N}V_{i}=\frac{\text{1}}{N}\sum_{i=\text{1}}^{N}\sum_{j=\text{1}}^{N}a_{ij} \end{array}$$ (1)

Clustering coefficient (C) [25,26] is a local property and denotes the likelihood that neighbors of a vertex will also be connected to each other.


$$\begin{array}{c} C=\;\frac{\text{1}}{N}\sum_{i=\text{1}}^{N}\begin{array}{c} \underset{―}{\sum_{k\neq i}\sum_{\begin{array}{c} l\neq i\\ l\neq k \end{array}}a_{ik}a_{il}a_{kl}}\\ \sum_{k\neq i}\sum_{\begin{array}{c} l\neq i\\ l\neq k \end{array}}a_{ik}a_{il} \end{array} \end{array}$$ (2)

Characteristic path length (L) [25–27] is the average of the shortest path between pairs of vertices. The shortest path length between electrodes i and j ($d_{ij}$) were calculated by the distances function (in the igraph package) in R.


$$\begin{array}{c} L=\frac{\text{1}}{N(N-\text{1})}\sum_{i,j\in N,i\neq j}d_{ij} \end{array}$$ (3)

Efficacy (E) [27] is based on reciprocal of characteristic path length.


$$\begin{array}{c} E=\frac{\text{1}}{N(N-\text{1})}\sum_{i,j\in N,i\neq j}\frac{\text{1}}{d_{ij}} \end{array}$$ (4)

Small-worldness (SW) [25,27,28] measures the efficiency of information transmission.


$$\begin{array}{c} SW=\frac{C/C_{rand}}{L/L_{rand}} \end{array}$$ (5)

$C_{rand}$, and $L_{rand}$ represents C and L in random network, respectively. A random network is a network in which $a_{ij}$ is randomly rearranged from the original network. C and L were calculated in 300 random networks and their averaged values were denoted as $C_{rand}$ and $L_{rand}$.

Modularity (Q) [29,30] is a metric that quantifies the quality of dividing a network into several non-overlapping modules. Here, network is composed of nodes and edges. In this study, Nodes and edges correspond to electrodes where EEGs were recorded and $SL_{ij}$ or $exp(Z(SL_{ij}))$, respectively. In terms of the quality of network partitioning, high modularity is defined that nodes within the same module are tightly connected, while nodes between different modules are loosely connected. For mathematical details on the calculation of modularity, see [29]. Modularity was calculated by the netcarto function (in the rnetcarto package) in R.

In eigenvector centrality ($x_{eigen}$) [31], the centrality of a unit is the sum of its connectivity to other units and weighted by the centrality of those other units. Eigenvector centrality was calculated by the evcent function (in the igraph package) in R.


$$\begin{array}{c} {\lambda x}_{\text{eigen}}=Ax_{\text{eigen}} \end{array}$$ (6)

Where λ is maximum eigenvalue of matrix A.

Hub centrality ($x_{eigen}$) is determined based on outgoing links, while authority centrality ($x_{eigen}$) is determined based on incoming links [32,33].


$$\begin{array}{c} {A^{\text{T}}Ax}_{\text{hub}}=\;\lambda x_{\text{hub}} \end{array}$$ (7)

$$\begin{array}{c} {{AA}^{\text{T}}x}_{\text{auth}}=\;\lambda x_{\text{auth}} \end{array}$$ (8)

Where λ is maximum eigenvalue of matrix A. In this study, $A^{T}A=AA^{T}$ because A is undirected and symmetric matrix. We just focused on hub centrality. Hub centrality was calculated by the hub.score function (in the igraph package) in R.

Closeness centrality ($x_{closen}$) [34] takes an approach by the shortest path length. This centrality is based on the idea that “Units that can reach other units via the shortest possible path are central.” Closeness centrality was calculated by the closeness function (in the igraph package) in R.


$$\begin{array}{c} x_{\text{closen}}(i)=\frac{\text{1}}{\sum_{j=\text{1}}^{N}d_{i,j}} \end{array}$$ (9)

PageRank ($x_{page}$) [34–36] was originally suggested as one of the methods for evaluating web pages on the World Wide Web (WWW). PageRank was calculated by the page.rank function (in the igraph package) in R.


$$\begin{array}{c} x_{\text{page}}(i)=(\text{1}-\alpha )\frac{\text{1}}{N}+\alpha \sum_{j=\text{1}}^{N}{\rho_{ij}\times x}_{\text{page}}(j) \end{array}$$ (10)

$$\begin{array}{c} \rho_{ij}=\frac{a_{ij}}{\text{max}\left(\sum_{h=\text{1}}^{N}a_{jh},\text{1}\right)} \end{array}$$ (11)

Where α is adjustment parameter (α=0.85) and $\rho_{ij}$ is probability transition matrix in electrodes i and j. The four types of centralities mentioned above were calculated for each brain region.

### 2.3. Construction of classifiers

**Fig 1:**
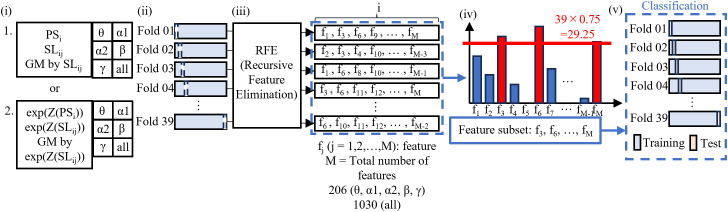
Outline of the construction of feature subsets. (i) 1. is Feature set 1 ($PS_{i}$, $SL_{ij}$, and graph metrics (GM) by $SL_{ij}$), and 2. is Feature set 2 ($exp(Z\left(PS_{i}\right))$, $exp(Z(SL_{ij}))$, and graph metrics (GM) by $exp(Z(SL_{ij}))$). Feature sets were constructed for each frequency band and for all frequency bands combined. The number of features was 16+120+70=206 dimensions for each frequency band, and 206×5=1030 dimensions for all frequency bands combined. (ii) Construction of leave-one-out datasets. The number of folds is equal to the number of subjects. (iii) Feature selection by RFE. In each fold, number of i features were selected from number of *M* features. M was 206 for each frequency band, and M was 1030 for all frequency bands combined. (iv) Counting the selection frequency of features. A set of features which selected in more than 75% of the folds, corresponding to 30 or more folds, was defined as the feature subset. (v) For the constructed feature subset, micro-F1 and macro-F1 score were calculated by LOOCV.

Classifiers were constructed to accurately classify the 3 subject groups. To calculate accuracy, feature subset, which was defined as a set of selected features, was constructed (Fig 1).

Features were selected from Feature set 1 ($PS_{i}$, $SL_{ij}$, and graph metrics by $SL_{ij}$) or Feature set 2 ($exp(Z\left(PS_{i}\right))$, $exp(Z(SL_{ij}))$, and graph metrics by $exp(Z(SL_{ij}))$). These feature sets are provided as S1 and S2 Files, respectively. Feature sets were constructed for each frequency band and for all frequency bands combined. The number of features was 16+120+70=206 dimensions for each frequency band, and 206×5=1030 dimensions for all frequency bands combined.

First, a leave-one-out dataset is constructed for feature selection. Since each subject was left out once, this consisted of 39 folds.

Second, number of i features were selected for each fold. Recursive Feature Elimination (RFE) was applied for the selection. RFE is a method that selects features using a machine learning algorithm. This method scans the set of features to identify which ones are important, and eliminates features until the specified number is reached. The features selected could vary depending on which algorithm is applied. 8 types of machine learning algorithms were used: decision tree, logistic regression, SVM (support vector machine), random forest, gradient boosting, XGBoost (eXtreme Gradient Boosting), AdaBoost (Adaptive Boosting), and ExtraTree (Extremely Randomized Trees).

Third, the features that were frequently selected were searched after feature selection was completed for the entire fold. We aggregated features that were selected in more than 75% of the folds, corresponding to 30 or more folds. Set of these features were defined as feature subset.

For the constructed feature subsets, micro-F1 and macro-F1 score were calculated by leave-one-out cross-validation (LOOCV). Micro-F1 score is the proportion of correct predictions out of all predictions. Macro-F1 score is the average of F1 score of 3 subject groups. LOOCV was performed by the same 8 types of machine learning algorithms as in RFE.

These steps were executed for the feature sets of each of 5 frequency bands, as well as for the feature set of all frequency bands combined. The construction of feature subsets was performed for all patterns of i, (1≤i≤206 when using the feature set of a single frequency band, and 1≤i≤1030 when using the set of all frequency bands). We searched for the classifier with the highest micro-F1 score among the classifiers that achieved accuracy of over 80% at all 3 subject groups.

We applied the SHAP (SHapley Additive exPlanations), a tool for interpreting machine learning models’ predictions. This tool generates individual-level feature importance scores known as SHAP values, which quantify the contribution of each feature to a specific prediction outcome [37]. In this study, SHAP was applied at each fold for LOOCV to examine the features that were particularly important in the feature subsets. SHAP values for each feature were the average of 39 folds.

As a supplementary evaluation, the classifier with the highest micro-F1 score was trained using all 39 subjects and then tested on the same subjects to assess apparent classification within the present dataset.

These machine learning analyses were performed using Python 3.12.3 with scikit-learn 1.8.0, xgboost 3.1.0, and SHAP 0.50.0. Unless otherwise specified, default hyperparameters implemented in each library were used (Table 1).

**Table 1:** Machine learning classifiers and implementation settings.

Algorithms	Implementation	Parameters
Decision tree	DecisionTreeClassifier	Max_depth = 3, random_state = 0
Logistic regression	LogisticRegression	random_state = 0
SVM	LinearSVC	random_state = 0
Random forest	RandomForestClassifier	random_state = 0
Gradient boosting	GradientBoostingClassifier	random_state = 0
XGBoost	XGBClassifier	random_state = 0
AdaBoost	AdaBoostClassifier	random_state = 0
ExtraTree	ExtraTreesClassifier	random_state = 0

### 2.4. Nested leave-one-out cross-validation

**Fig 2:**
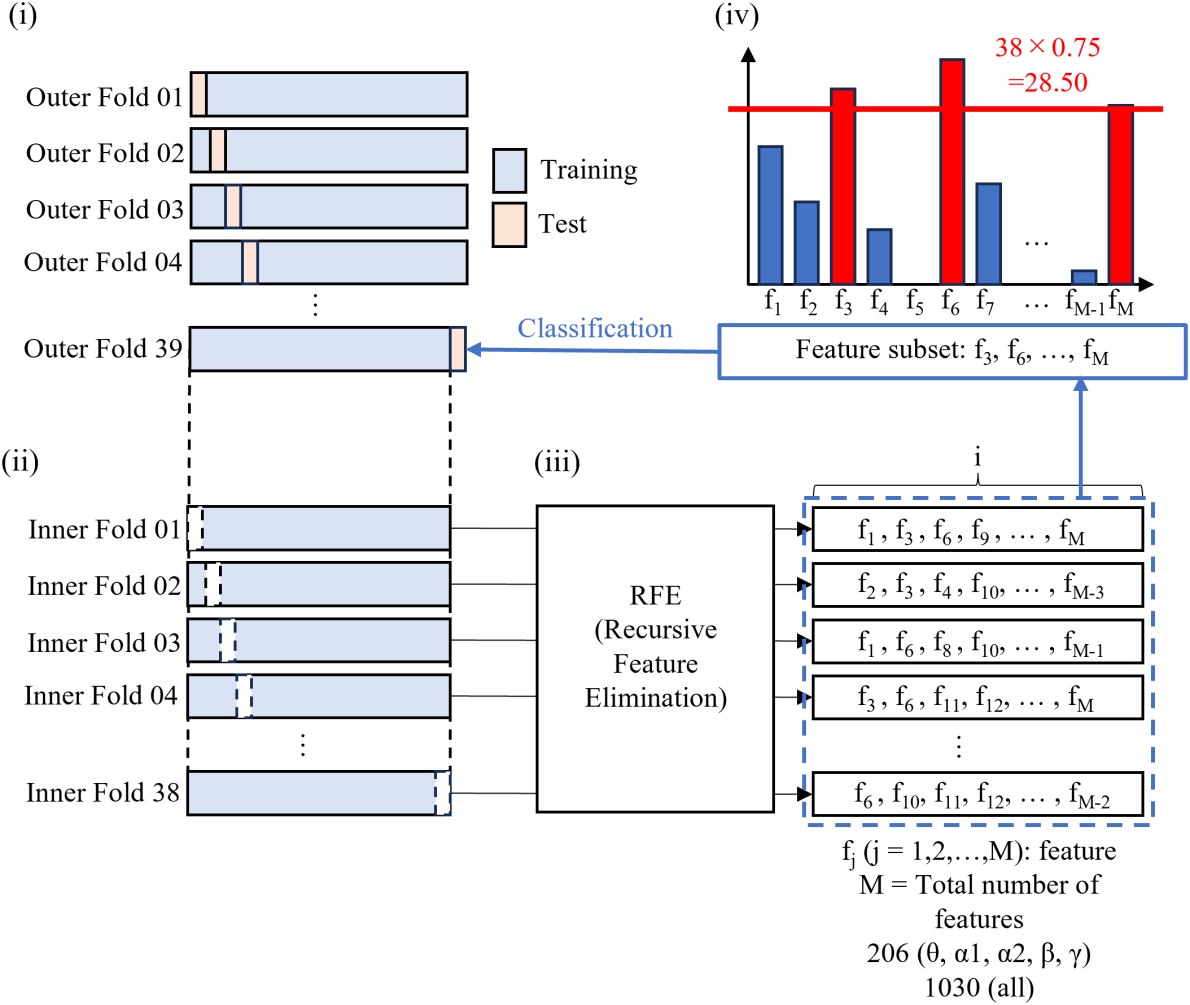
Outline of nested LOOCV at Outer Fold 39. (i) Construction of outer fold for outer cross-validation. 38 subjects were used as the training data and the remaining 1 subject used as the test data. (ii) Construction of inner fold for inner cross-validation using the 38 training subjects. The inner dataset was divided into 38 folds, with 37 subjects used for feature selection and 1 subject held out in each fold. RFE was applied for feature selection. In each fold, number of i features were selected from number of *M* features. M was 206 for each frequency band, and M was 1030 for all frequency bands combined. (iii) Counting the selection frequency of features. A set of features which selected in more than 75% of the inner folds, corresponding to 29 or more folds, retained as the feature set for that outer fold. (iv) Classification of outer test subject. The classifier was trained on 38 subjects in the outer training set and used to predict the classification of the outer test subject. These procedures were repeated across all 39 outer folds, and the overall classification performance was calculated from the predictions obtained for all subjects.

To evaluate classification performance while minimizing the risk of information leakage during feature selection, nested leave-one-out cross-validation (nested LOOCV) was performed using the feature set and the machine learning algorithms that constructed the classifier with the highest micro-F1 score in the primary analysis (Fig 2).

In the outer cross-validation, the dataset consisting of 39 subjects was divided into 39 folds, with 38 subjects used as the training data and the remaining 1 subject used as the test data in each fold. Within each outer training data, an inner LOOCV procedure was further conducted using the 38 training subjects. Specifically, the inner dataset was divided into 38 folds, with 37 subjects used for feature selection and 1 subject held out in each fold. RFE was applied independently to the 37 subjects in each inner fold. After completing all 38 inner folds, features selected more than 75% of the inner folds, corresponding to 29 or more folds, were retained as the feature set for that outer fold. By this selected feature set, the classifier was trained on all 38 subjects in the outer training set and then used to predict the classification of the outer test subject. This procedure was repeated across all 39 outer folds, and the overall classification performance was calculated from the predictions obtained for all subjects.

These steps were executed for the feature sets of the frequency bands indicated the highest micro-F1 score. The construction of feature subsets was performed for all patterns of i, (1≤i≤206 when using the feature set of a single frequency band, or 1≤i≤1030 when using the set of all frequency bands). Importantly, the outer test subject was completely excluded from all feature selection procedures in the corresponding fold.

### 2.5. Similarity-based subject removal analysis

The robustness of the classifiers with the highest micro-F1 score was evaluated. A group of subjects consisting of subjects who showed a high similarity with many others were determined, and a critical micro-F1 and macro-F1 score were calculated by LOOCV on a dataset with these subjects removed. To verify the similarity of the data, the Euclidean distance between all pairs of subjects for the feature subsets were calculated. The Euclidean distance data were sorted in ascending order, and the differences in Euclidean distances were calculated. To determine the group of subjects to be removed, we searched for points with large differences of Euclidean distance. As a method of search, we applied the IQR (Interquartile Range) formula using the first 50% of the data of difference of Euclidean distance.

The IQR formula is a way to detect outliers based on the interquartile range. Using the first quartile (Q1) and the third quartile (Q3), it is expressed by the following formula.


$$\begin{array}{c} IQR=Q\text{3}-Q\text{1} \end{array}$$ (12)

$$\begin{array}{c} (\text{Threshold})=Q\text{3}+\text{1}.\text{5}IQR \end{array}$$ (13)

Points where the difference of Euclidean distance changes significantly were assumed to be outliers, and data from pair of subjects with small Euclidean distances were extracted based on one of these points.

**Fig 3:**
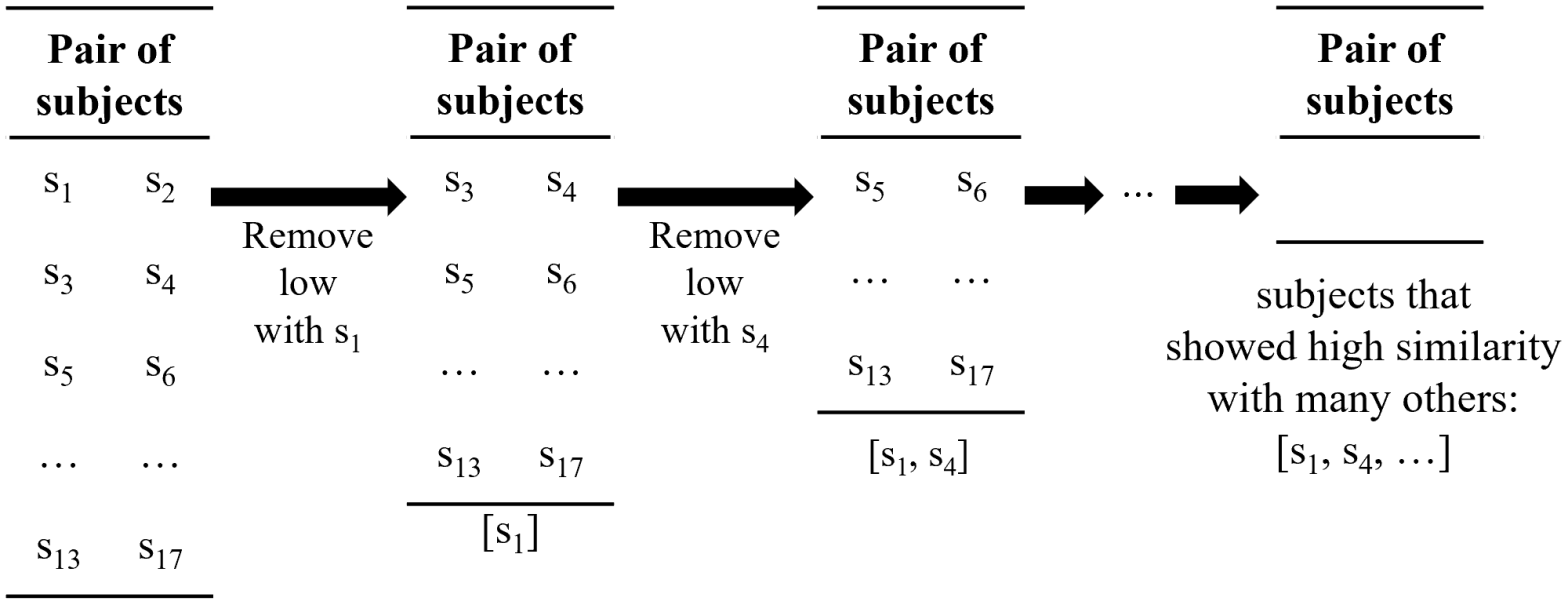
Outline for searching subjects that showed high similarity with many others from table for the extracted data of pair of subjects. For the pair of subjects with the smallest Euclidean distance (let $s_{\text{1}}$, $s_{\text{2}}$), the number of occurrences of each element within the table was counted. The subject with the higher count was selected as the data with higher similarity (let $s_{\text{1}}$), and all pairs including this data ($s_{\text{1}}$) were removed. Using the table after removing low with $s_{\text{1}}$, the same procedure was repeated for the pair of subjects with the smallest Euclidean distance. This process was continued until all data were removed from table.

For the extracted data of pair of subjects, subjects that showed high similarity with many others were identified (Fig 3). The group of subjects that showed high similarity with many others was removed from LOOCV, and critical classification accuracy was calculated.

### 2.6. Permutation test

To evaluate whether the observed classification performance could be obtained by chance, a permutation test was performed. In this analysis, the class labels were randomly shuffled while the feature matrix was kept unchanged, and the classification procedure was repeated by LOOCV. This process was repeated 10,000 times to generate a null distribution of classification performance under the assumption that there was no true relationship between indices and labels. The p-value was calculated as the proportion of permutation scores equal to or higher than the observed classification score. Permutation test supported that the observed performance of classifier with the highest micro-F1 score was unlikely to be reproduced under random label assignment.

Permutation test was performed using the scikit-learn library in Python 3.12.3. Permutation test was conducted using the permutation_test_score function implemented in sklearn.model_selection. The random seed was fixed to 0 throughout these analyses to ensure reproducibility.

### 2.7. Repeated stratified 10-fold cross-validation

To further assess the robustness and stability of the classifier, repeated stratified 10-fold cross-validation was performed. In stratified 10-fold cross-validation, the dataset is divided into 10 folds while preserving the proportion of each diagnostic class in each fold. This procedure was repeated multiple times with different random splits, and the mean and standard deviation of the classification performance were calculated. This analysis was used to evaluate whether the classifier performance was stable across different data partitions. Repeated stratified 10-fold cross-validation was performed by the classifier with the highest micro-F1 score identified in the primary analysis.

Repeated stratified 10-fold cross-validation was performed using the RepeatedStratifiedKFold class implemented in sklearn.model_selection. The random seed was fixed to 0 throughout these analyses to ensure reproducibility.

### 2.8. Use of artificial intelligence tools

During the preparation of this manuscript, the authors used ChatGPT (OpenAI) to assist with language editing, improving readability and responses to reviewer comments. The tool was not used to generate research data, perform statistical analyses, or make scientific conclusions. All AI-assisted text was reviewed, edited, and approved by the authors, who take full responsibility for the final content of the manuscript.

## 3. Results

### 3.1. Classifier with high accuracy

**Fig 4:**
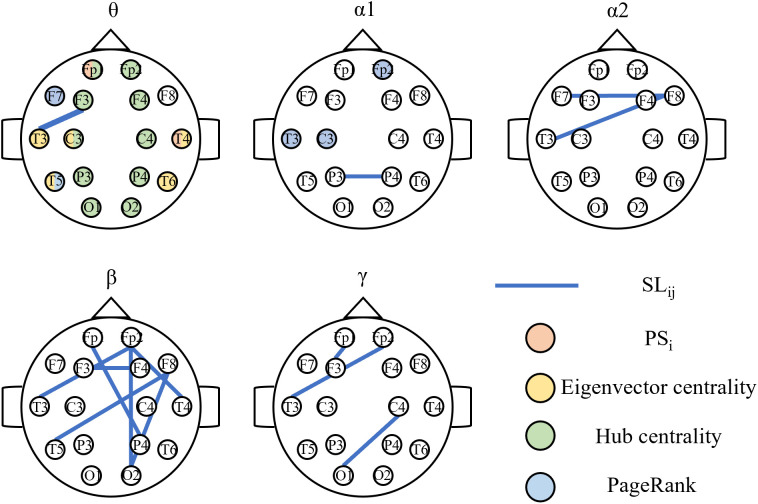
Feature subset in Classifier 1. This feature subset consisted of 36-dimensional features. Blue lines represent ${SL}_{ij}$. Orange, yellow, green, and blue circles represent ${PS}_{i}$, eigenvector centrality, hub centrality, and PageRank, respectively.

**Fig 5:**
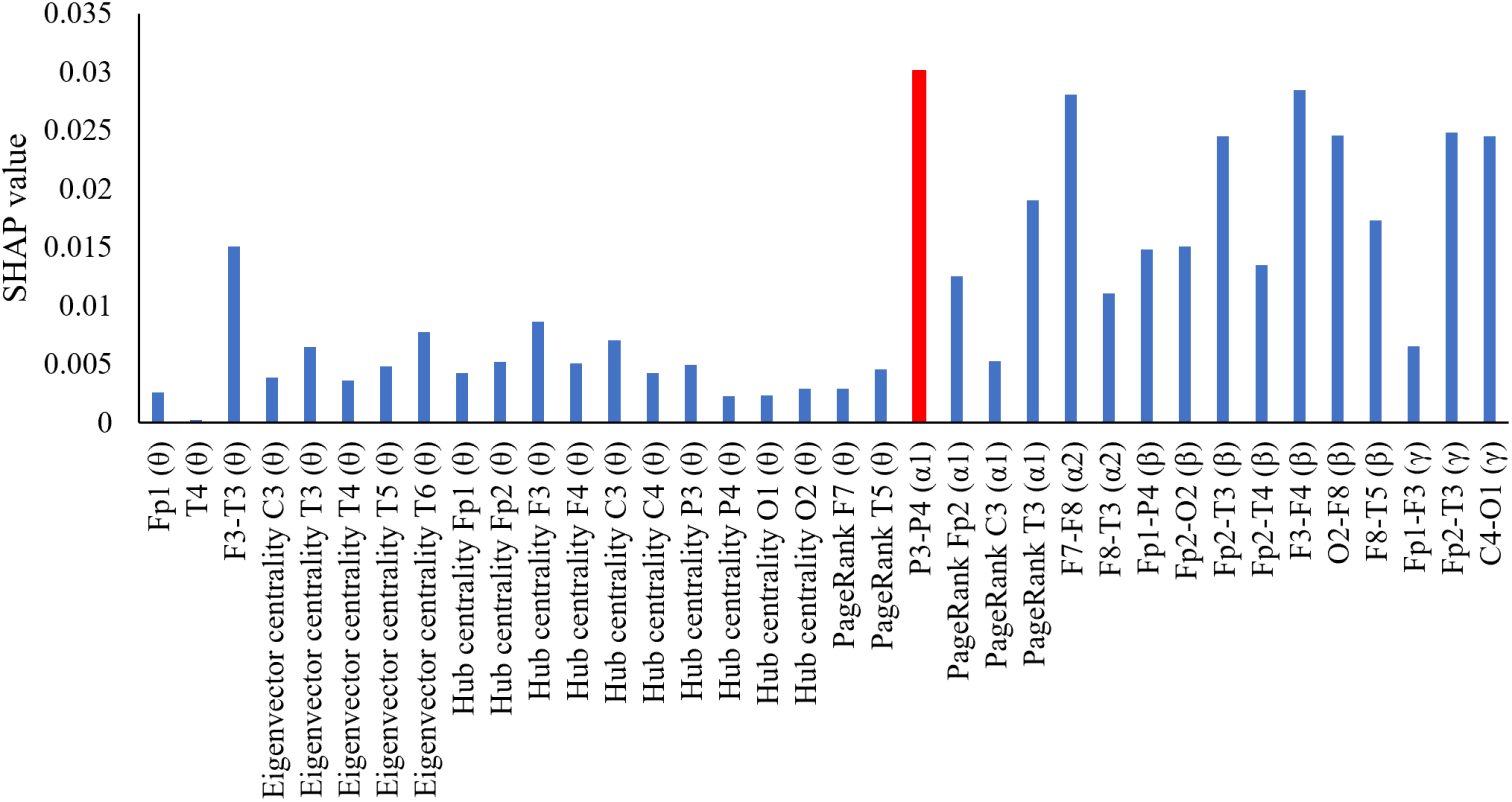
SHAP values of each feature in feature subset in Classifier 1. The highest SHAP value was $SL_{ij}$ between P3 and P4 for lower alpha band (red bar).

In the classifier based on Feature set 1, the highest micro-F1 score was 92.31% (Healthy older adults: 80.00%, AD patients: 100.00%, sCJD patients: 83.33%). Macro-F1 score of this was 91.23%. Feature subset consisted of 36-dimension (Classifier 1) (Fig 4, Table 2). In this classifier, feature selection by RFE and LOOCV were conducted by XGBoost. As a result of calculating feature importance by SHAP, the highest SHAP value was observed in $SL_{ij}$ between P3 and P4 for lower alpha band (Fig 5).

**Table 2:** Feature subset in Classifier 1.

Frequency band	Feature subset		
	${PS}_{i}$	${SL}_{ij}$	Graph Metrics
θ	Fp1, T4	F3-T3	Eigenvector centrality C3 Eigenvector centrality T3 Eigenvector centrality T4 Eigenvector centrality T5 Eigenvector centrality T6 Hub centrality Fp1 Hub centrality Fp2 Hub centrality F3 Hub centrality F4 Hub centrality C3 Hub centrality C4 Hub centrality P3 Hub centrality P4 Hub centrality O1 Hub centrality O2 PageRank F7 PageRank T5
α1	–	P3-P4	PageRank Fp2 PageRank C3 PageRank T3
α2	–	F7-F8, F8-T3	–
β	–	Fp1-P4, Fp2-O2, Fp2-T3, Fp2-T4, F3-F4, O2-F8, F8-T5	–
γ	–	Fp1-F3, Fp2-T3, C4-O1	–

**Fig 6:**
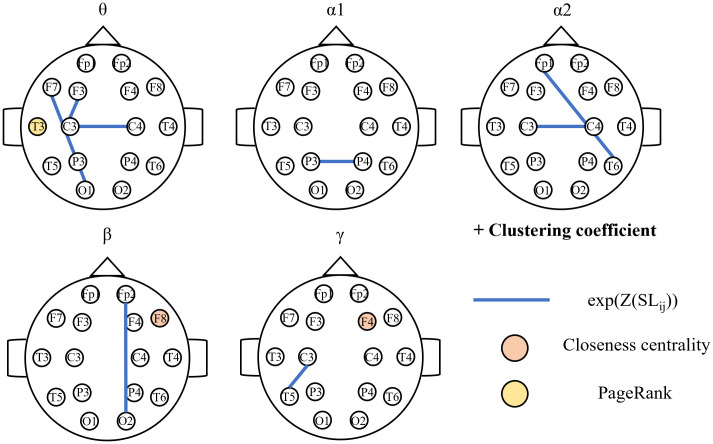
Feature subset in Classifier 2. This feature subset consisted of 12-dimensional features. Blue lines represent $exp(Z(SL_{ij}))$. Orange and yellow circles represent closeness centrality and PageRank, respectively.

**Fig 7:**
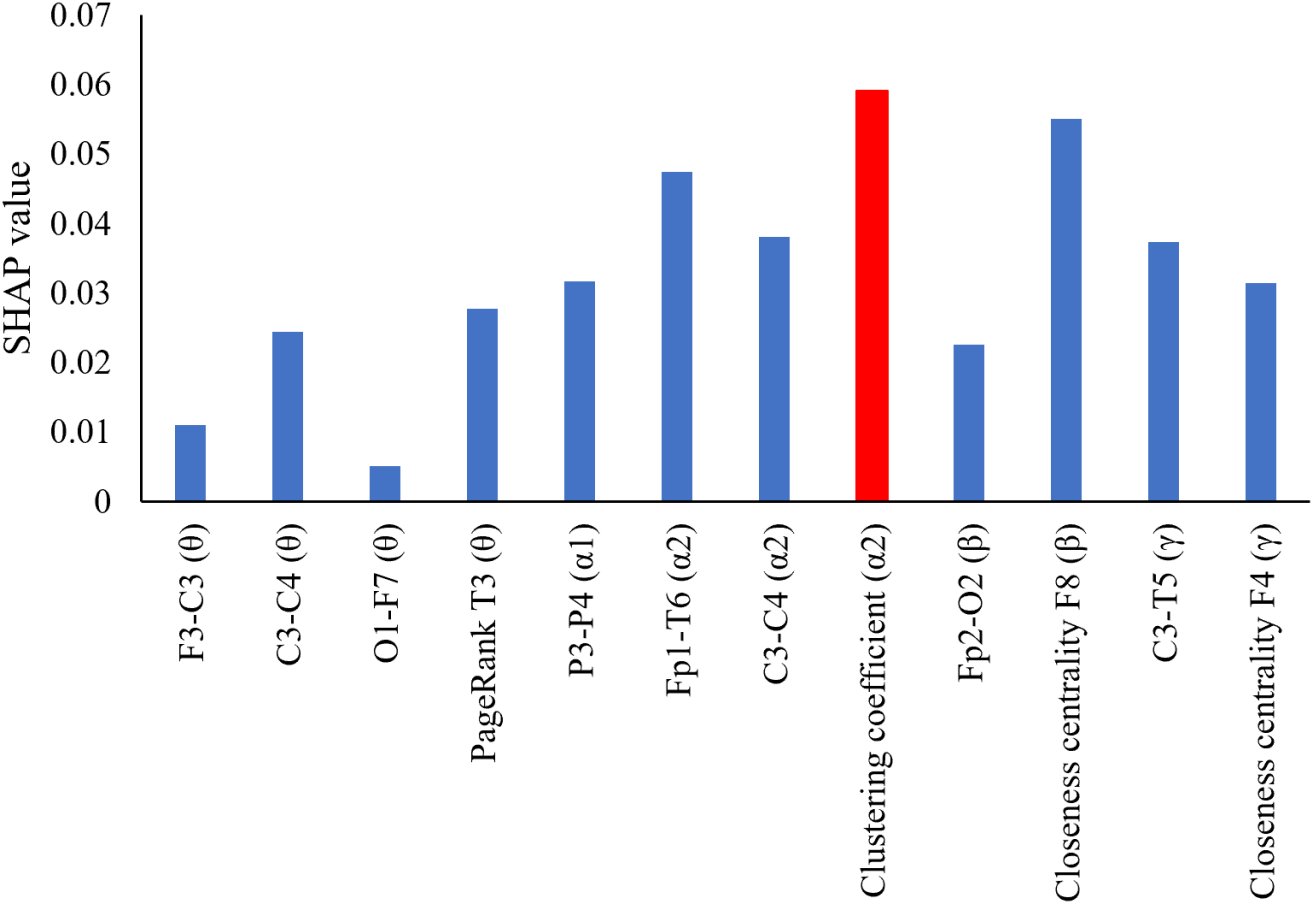
SHAP values of each feature in feature subset in Classifier 2. The highest SHAP value was clustering coefficient for upper alpha band (red bar).

In the classifier based on Feature set 2, the highest micro-F1 score was 97.44% (Healthy older adults: 90.00%, AD patients: 100.00%, sCJD patients: 100.00%). Macro-F1 score of this was 97.54%. Feature subset consisted of 12-dimension (Classifier 2) (Fig 6, Table 3). In this classifier, features were selected by RFE with XGBoost and LOOCV were conducted by AdaBoost. As a result of calculating feature importance by SHAP, the highest SHAP value was observed in clustering coefficient for upper alpha band (Fig 7).

**Table 3:** Feature subset in Classifier 2.

Frequency band	Feature subset		
$exp(Z({PS}_{i}))$	$exp(Z(SL_{ij}))$	**Graph Metrics**
θ	–	F3-C3, C3-C4, O1-F7	PageRank T3
α1	–	P3-P4	–
α2	–	Fp1-T6, C3-C4	Clustering coefficient
β	–	Fp2-O2	Closeness centrality F8
γ	–	C3-T5	Closeness centrality F4

As a supplementary evaluation, Classifier 2 was trained using all 39 subjects and then tested on the same 39 subjects, respectively. In this evaluation, micro-F1 score of this classifier was 100.00%.

### 3.2. Nested leave-one-out cross-validation

To further evaluate the classification framework under a stricter leakage-controlled setting, nested LOOCV was performed. Feature selection was conducted using Feature set 2 for all frequency bands combined, XGBoost-based RFE within the training data of each outer fold, and classification was performed by AdaBoost.

Under this nested LOOCV, micro-F1 score of the classifier achieved was 84.62% (Healthy older adults: 60.00%, AD patients: 95.65%, sCJD patients: 83.33%). This score was observed when 26 features were selected in each fold by RFE. Among the 10 healthy older adults, 4 were misclassified as AD. Among the 23 AD patients, 1 was misclassified as a healthy control. Among the 6 sCJD patients, 1 was misclassified as a healthy older adult.

**Fig 8:**
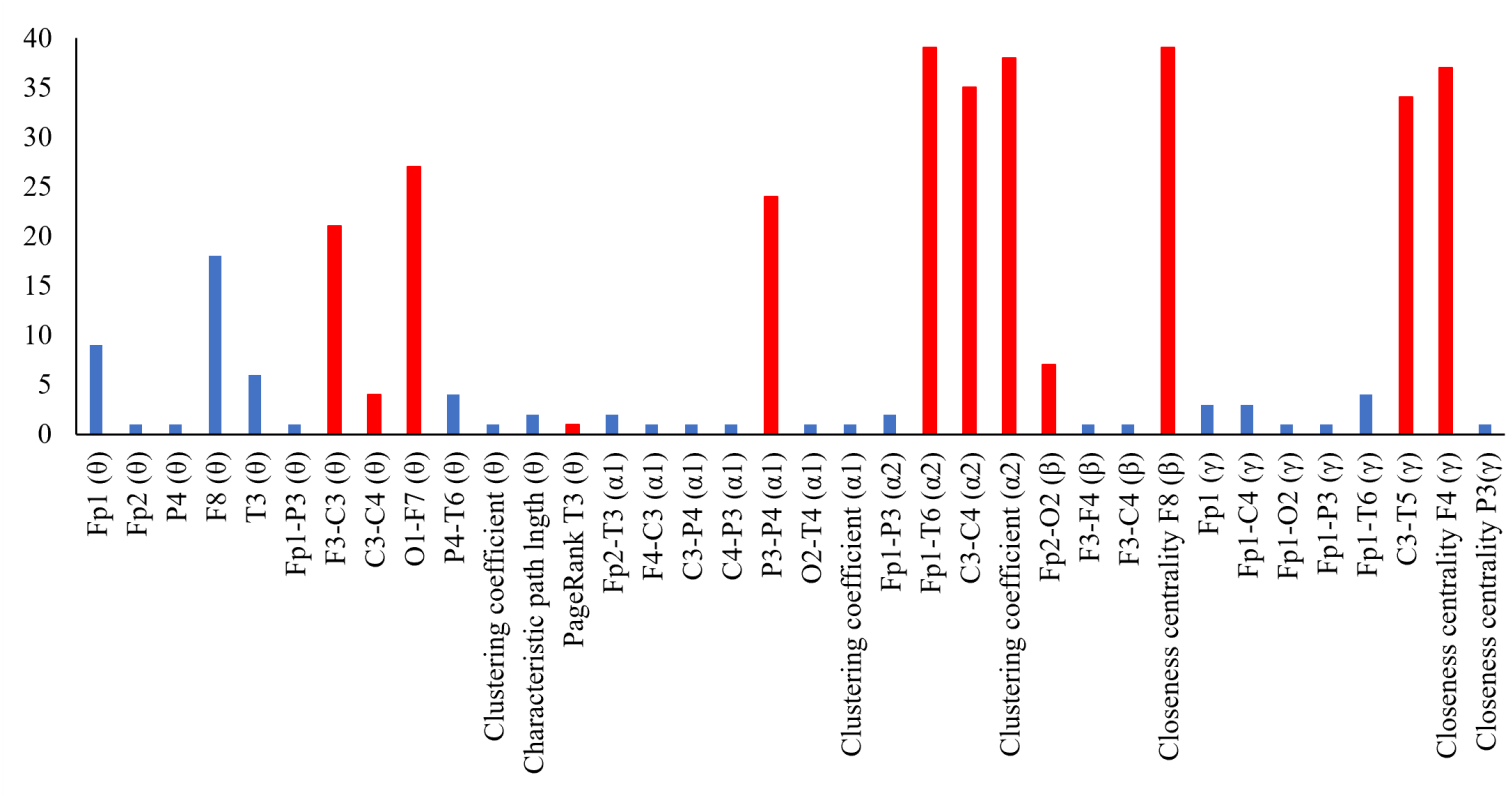
The frequency of the features selected for the feature subset. The x-axis represents the features selected in the feature subset, and the y-axis represents the frequency, meaning in how many outer folds each feature was selected. The red bars represent the features selected as the feature subset of Classifier 2.

The number of features retained after applying the more than 75% inner fold selection criterion varied across outer folds. The mean number of selected features was 9.56 ± 1.55. Fig 8 shows the frequency of the features selected for the feature subset.

### 3.3. Similarity-based subject removal analysis

We searched for subjects who showed high similarity with many others in the classifiers. The number of subjects used in the calculation of critical classification micro-F1 and macro-F1 score are shown in the Table 4.

**Table 4:** Number of subjects used in the calculation of critical micro-F1 and macro-F1 score.

Classifier	Healthy older adults	AD patients	sCJD patients
**1**	7	18	6
**2**	9	16	6

In the Classifier 1, calculation of micro-F1 and macro-F1 score was conducted by LOOCV with XGBoost. The critical micro-F1 score was 74.19% (Healthy older adults: 42.86%, AD patients: 83.33%, sCJD patients: 83.33%). Macro-F1 score was 70.76%.

In the Classifier 2, In this classifier, Feature selection by RFE and LOOCV were conducted by AdaBoost. The critical micro-F1 score was 90.32% (Healthy older adults: 77.78%, AD patients: 93.75%, sCJD patients: 100.00%). Macro-F1 score was 91.09%.

### 3.4. Permutation test

To evaluate whether the observed classification performance was significantly higher than that expected by chance, a permutation test was performed. In Classifier 2, micro-F1 score was 97.44%, whereas the mean permutation score was 45.00%. The observed score was significantly higher than the permutation-based null distribution (p-value < 0.0001). These indicate that the classification performance was unlikely to have been obtained by chance.

### 3.5. Repeated stratified 10-fold cross-validation

To further assess the stability of the classifier across different data partitions, repeated stratified 10-fold cross-validation was performed. In Classifier 2, a mean micro-F1 score was 92.21 ± 13.11% across repeated cross-validation runs. These results indicate that Classifier 2 maintained higher average performance across different stratified data splits, although some variability in performance was observed.

## 4. Discussion

In this study, power spectrum, SL values, and graph metrics were calculated from EEG data. The aim of this study is to construct a high accuracy classifier for the early and accurate diagnosis of sCJD. The differentiation not only from healthy older adults but also from Alzheimer’s disease patients who are representative cases of cognitive impairment associated with brain disorders was also the aim. The results of this study demonstrated a high classification accuracy for classifying sCJD patients from both healthy older adults and Alzheimer’s disease patients. Additionally, the robustness of classifier was evaluated. The most important finding of this study is that standardization and exponential transformation of indices and construction of various classifiers contributed to constructing the highest accuracy classifier.

High classification accuracy (micro-F1 score) was confirmed. The accuracy was comparable to prior studies on the classification of sCJD and non-sCJD using biomarkers other than EEG data (diffusion MRI [6], or RT-QuIC [7]). Although direct comparison with previous studies is difficult because of differences in cohorts, diagnostic tasks, and validation strategies, the results of this study suggested that EEG may provide useful information for classification of sCJD.

In diagnosis of sCJD patients, it is important to make distinctions not only from healthy controls of the same age but also from patients with other neurological disorders because sCJD patients present with various symptoms [1,4]. Morabito et al. [21] mainly conducted EEG-based CJD classification study, which separate binary classifiers for CJD versus RPD, AD, or healthy controls using CWT-derived features and deep learning representation. Our study attempted a single three-class classification framework for healthy older adults, AD patients, and sCJD patients. In addition, the classifier with the highest micro-F1 score was based on synchronization likelihood and graph metrics, which are directly interpretable in terms of brain organization. Although the sample size of the present study was smaller, especially for sCJD patients, the selected classifier was supported by nested LOOCV, similarity-based subject removal analysis, permutation test, and repeated stratified 10-fold cross-validation. It suggested that the observed performance was not solely attributable to chance-level associations or a single favorable data partition.

To investigate the usefulness of standardization and exponential transformation of indices for high classification accuracy, Classifier 1 and 2 were compared. The highest micro-F1 score by Classifier 2 was 97.44% (Healthy older adults: 90.00%, AD patients: 100.00%, sCJD patients: 100.00%). On the other hand, the highest micro-F1 score by Classifier 1 was 92.31% (Healthy older adults: 80.00%, AD patients: 100.00%, sCJD patients: 83.33%).

To construct high accuracy classifiers, various feature subsets were constructed. Feature set for each frequency band (5 frequency bands) and all frequency bands combined were set. Additionally, the number of features selected by RFE was sequentially increased from 1 to 206 for each frequency band (from 1 to 1030 when using the feature set from all frequency bands). Moreover, the features selected differ depending on the algorithm which applied to RFE. Therefore, for Feature set 1 or 2, it was theoretically possible to construct up to 5×206×8+1030×8=16,480 feature subsets. In this study, fewer than 16,480 feature subsets were constructed. In this study, we were able to construct a classifier with an accuracy of 97.44% (Classifier 2). This classifier indicated the accuracy for each subject group exceeding 90%. It was concluded that generating various feature subsets contributed to the construction of the Classifier 2.

Focusing on the number of features of classifiers, Classifier 2 had fewer features compared to Classifier 1. However, it demonstrated higher classification accuracy. In addition, power spectrum was not selected as features for Classifier 2. SL values and graph metrics were selected exclusively.

In the supplementary evaluation, Classifier 2 was trained using all 39 subjects and then tested on the same 39 subjects, respectively. In this evaluation, micro-F1 score was 100.00%. This result should not be regarded as evidence of generalization performance because the risk of overfitting remains. Given the small sample size and the high dimensional-feature of the EEG-derived indices, this finding should be interpreted cautiously. In addition, the results suggest that the ability of classifier to generalize to unseen data may be limited and that the classifier may have partially learned dataset-specific characteristics.

Nested LOOCV was additionally performed under a stricter validation framework in which feature selection was confined to the training data of each outer fold. Under this leakage-controlled condition, the XGBoost-based RFE and micro-F1 score was calculated by AdaBoost using Feature set 2 for all frequency bands combined. The highest micro-F1 score was 84.62%. Although this score was lower than that observed in primary study, the classifier retained meaningful classification ability when feature selection was performed independently within each outer fold.

The class-wise results showed high classification accuracy for AD patients (95.65%) and sCJD patients (83.33%), whereas the accuracy for healthy older adults was relatively lower (60.00%). Most misclassified healthy older adults were predicted as AD. It suggested that indices selected under the nested LOOCV may capture disease-related alterations more effectively than differences between healthy aging and AD-related changes. Importantly, the nested LOOCV procedure retained a compact set of features across outer folds, with an average of 9.56 ± 1.55 selected features. This feature subset size was comparable to that of the primary classifier. This result suggested that the result of nested LOOCV was not achieved by relying on an excessively large number of features. Rather, a relatively small subset of EEG-derived indices appeared to contain discriminative information for classification of sCJD, AD, and healthy older adults. In addition, Fig 8 showed that the features frequently selected by nested LOOCV were similar with those selected in Classifier 2. This suggested that the selected features were relatively stable.

The reduction in accuracy from the primary analysis to nested LOOCV suggests that the original performance estimate may have been partially optimistic, which is an important consideration in small-sample, high-dimensional classification studies. Nevertheless, the nested LOOCV result supports the potential utility of EEG-derived indices for classification.

Evaluation of robustness was conducted on the classifiers with the highest micro-F1 score. As an evaluation method, subjects that show high similarity with many subjects were removed from the original subject data. After this removal, LOOCV was performed on the remaining subject data after this removal. The accuracy in this LOOCV was used as a critical micro-F1 and macro-F1 score for evaluation of robustness. When there are high-similarity subject data, the test data is more susceptible to the influence of high-similarity training data. If these high-similarity data belong to the same subject group, they are likely to contribute to high classification accuracy and to leave the possibility of overfitting. Therefore, high-similarity data were removed in this method. As a result, a high critical micro-F1 score was confirmed by Classifier 2. Micro-F1 score was 90.32%. On the other hand, a high critical micro-F1 score wasn’t confirmed by Classifier 1. Micro-F1 score was 74.19%. Therefore, these results suggest that standardized and exponentially transformed indices calculated from EEG may be useful for classification among healthy older adults, AD patients, and sCJD patients. This analysis also suggests that Classifier 2 was not solely driven by highly similar subjects.

Additional validation analyses were performed to evaluate the robustness and statistical reliability of Classifier 2. Permutation test showed that the observed classification performance was significantly higher than the performance obtained after random shuffling of class labels. This result suggests that the classifier did not merely reflect chance-level associations between EEG features and diagnostic labels. In addition, repeated stratified 10-fold cross-validation demonstrated relatively stable classification performance across different data partitions. These findings support the robustness of the classifier and suggest that the selected EEG-derived indices may capture disease-related alterations in brain organization.

To assess the generalizability of Classifier 2, external validation was performed using an independent EEG dataset. This EEG dataset was not used for feature selection, model training, or internal cross-validation. The external validation dataset consisted of 2 sCJD patients. The EEG recordings in the external validation dataset were acquired under conditions similar with those of the internal dataset in several aspects. The sampling frequency was the same (500 Hz), electrodes were attached based on the international 10–20 system, and resting-state EEG recordings were obtained with eye close. However, the low-pass filter setting differed (70 Hz), and no notch filter was applied in the external validation dataset. In addition, details of the measurement location and measuring equipment were withheld. The same frequency band, calculation of features of EEG-derived indices were applied. Classifier 2 was trained using the internal dataset with the algorithm indicated the highest micro-F1 score and subsequently applied to the external validation dataset without further modification. Classification was evaluated micro-F1 and macro-F1 score.

In the validation dataset, micro-F1 and macro-F1 score of Classifier 2 were 0.00%. These indicated that none of the cases were correctly predicted in this dataset. These results suggested that the performance observed during internal validation was not generalizable to the external cohort. Possible explanations include differences in heterogeneity in patient characteristics or measurement conditions, limited sample size, and potential overfitting to the internal dataset. Therefore, the external validity of Classifier 2 is insufficient.

This study identified synchronization likelihood- and graph metrics-based features in the upper alpha band as important contributors to the classification. The evaluation of feature importance by SHAP revealed that clustering coefficient for upper alpha band contributed the most in Classifier 2. Previous studies have suggested that alpha-band oscillatory activity is closely associated with large-scale cortical communication, attention, and semantic memory processing [38–40]. Therefore, synchronization likelihood- and graph metrics-based feature for upper alpha band may reflect impaired neuronal communication associated with neurodegenerative processes.

Prion diseases are characterized by progressive synaptic dysfunction, neuronal loss, spongiform degeneration, and abnormal prion protein accumulation [41]. All of them would contribute to widespread disruption of brain network organization. Similarly, Alzheimer’s disease has been associated with impaired functional connectivity, and alterations in small-world network organization [25]. EEG and MEG studies have consistently demonstrated that neurodegenerative disorders are accompanied by disturbances in large-scale brain networks [42]. Previous studies have suggested that alterations in EEG synchronization and functional brain network organization are closely associated with cognitive decline in neurodegenerative disorders. Pijnenburg et al. reported that synchronization likelihood in the upper alpha band was significantly reduced in patients with Alzheimer’s disease and mild cognitive impairment during a working memory task, and that cognitive task performance was strongly associated with global cognitive status assessed by MMSE [43]. Stam et al. have also shown reduced EEG synchronization in Alzheimer’s disease and mild cognitive impairment, with altered synchronization patterns being associated with cognitive impairment severity [44].

It is possible that the oscillations for upper alpha band are associated with the cognitive state related to sCJD. For alpha band, the differences of some indices were reported in various previous studies (MCI [45], epilepsy [46], perinatal stroke [47], Parkinson’s disease (PD) [48], and bipolar affective disorder [49]). These findings support the interpretation that synchronization likelihood and graph metrics for upper alpha band identified in this study may reflect dysfunction of large-scale cortical integration mechanisms.

Graph theoretical analysis has also been widely used to characterize pathological alterations in brain network topology in neurodegenerative disorders [50]. Previous studies have demonstrated that AD is associated with reduced network efficiency, disrupted small-world organization, and altered connectivity hub structure [25]. Because synchronization likelihood reflects statistical interdependence between EEG signals, altered synchronization likelihood in the upper alpha band may indicate abnormal large-scale neuronal coordination caused by neurodegenerative damage. Furthermore, network neuroscience frameworks propose that many neurological disorders can be understood as disorders of brain network organization [42]. From this perspective, the observed alterations in graph metrics for upper alpha band in sCJD may reflect widespread network disintegration associated with rapidly progressive neurodegeneration. However, because the present study did not include neuropathological confirmation or detailed molecular subtype information, the precise biological mechanisms underlying the observed EEG alterations remain unclear. Additional studies combining EEG connectivity analysis with clinical, molecular, and neuropathological assessments will be necessary to further clarify the pathophysiological significance of upper alpha network disruption in sCJD.

The main limitations are that the sample size is small and there is an imbalance in the number of subjects depending on the subject group, particularly the limited number of sCJD patients. It is true that additional EEG datasets are needed. Although sCJD is an extremely rare disease, the small sample size may limit statistical power and increase the risk of overestimating classification performance. Therefore, independent cohort validation and/or multi-center data aggregation would be essential to establish the robustness and generalizability of the proposed classifier in future studies.

However, this study proposes a framework for construction of classifiers by features calculated from EEG data. This study aims to distinguish between healthy older adults and Alzheimer’s disease, a representative example of cognitive dysfunction-related brain diseases, but it also focuses on evaluating the classification possibilities with EEG data from other brain disease patients, thereby contributing to high accuracy sCJD diagnosis.

In addition, the present study did not explicitly account for patient-specific clinical conditions, such as disease stage, symptom severity, progression rate, molecular subtype, or other individual pathological differences. Because both sCJD and Alzheimer’s disease exhibit substantial biological and clinical heterogeneity, EEG patterns may vary considerably among patients. Therefore, the proposed classifier may not fully capture the diversity of electrophysiological characteristics across individuals and may instead partially reflect features specific to a limited subset of patients included in this cohort. This limitation is particularly important given the very small number of sCJD cases analyzed in this study.

## 5. Conclusion

The aim of this study was to construct a classifier with high accuracy for classification of sCJD patients from AD patients and healthy older adults using EEG-derived indices. The highest classification accuracy in the primary analysis was 97.44%. It achieved using a 12-dimensional feature subset selected from standardized and exponentially transformed values. In addition, nested LOOCV, similarity-based subject removal analysis, permutation test, and repeated stratified 10-fold cross-validation supported that the classifier with the highest accuracy retained meaningful classification ability within the present dataset. In particular, the accuracy of nested LOOCV was 84.62% under a leakage-controlled validation framework. These findings suggest that resting-state EEG-derived indices may be useful candidate features for sCJD classification. However, further validation using larger, independent, and multi-center cohorts is required to establish the generalizability and clinical applicability of the proposed classifier.

## Supporting information

S1 File Feature set 1. De-identified feature set. This dataset contains the de-identified EEG-derived indices before standardization and exponential transformation. The indices include power spectrum, SL values, and graph metrics calculated across frequency bands. Each row corresponds to one subject, and each column corresponds to one index. No directly identifiable personal information is included.
(XLSX)

S2 File Feature set 2. De-identified feature set. This dataset contains the de-identified EEG-derived indices after standardization and exponential transformation. The indices include power spectrum, SL values, and graph metrics calculated across frequency bands. Each row corresponds to one subject, and each column corresponds to one index. No directly identifiable personal information is included.
(XLSX)
